# Antibacterial and antioxidant effect of ethanol extracts of *Terminalia chebula* on *Streptococcus mutans*

**DOI:** 10.1002/cre2.467

**Published:** 2021-06-28

**Authors:** Yea Ji Nam, Young Sun Hwang

**Affiliations:** ^1^ Department of Biomedical Laboratory Science, College of Health Sciences Eulji University Seongnam Republic of Korea; ^2^ Department of Dental Hygiene, College of Health Science Eulji University Seongnam Republic of Korea

**Keywords:** dental caries, ethanol extract of *Terminalia chebula*, plaque, *Streptococcus mutans*

## Abstract

**Objective:**

Dental caries is a high prevalent chronic bacterial infectious disease caused by plaque, a bacterial colony deposited on tooth surfaces and gum tissues. *Streptococcus mutans* is a primary cariogenic bacterium commonly found in the human oral cavity. Oral hygiene products containing antibacterial ingredients can be helpful in caries management. In this study, we investigated the anticaries mechanism of the ethanol extract of *Terminalia chebula* (EETC) on *S. mutans* and suggest its possible application as a functional ingredients for oral hygiene products.

**Materials and methods:**

The EETC was prepared from the *Terminalia chebula* fruit. Disk diffusion, minimum inhibitory concentration (MIC), minimum bactericidal concentration (MBC), and colony forming unit (CFU) were analyzed to observe the antibacterial activity of EETC. The glucan formation was measured using the filtrate of bacterial culture medium and sucrose. Gene expression was analyzed using RT‐PCR. Cytotoxicity was analyzed using the MTT assay. The radical‐scavenging activities of DPPH and ABTS were also tested to verify the antioxidant activity of EETC.

**Results:**

The antibacterial activity of the EETC was explored through a disc diffusion analysis and CFU measurement. EETC treatment decreased insoluble glucan formation and gene expression of glycosyltransferase B (*gtf B*), glycosyltransferase C (*gtf C*), glycosyltransferase D (*gtf D*), and fructosyltransferase (*ftf*). The MIC and MBC of EETC on *S. mutans* were not cytotoxic to gingival fibroblasts. In addition, we observed DPPH and ABTS‐radical scavenging activities of EETC.

**Conclusions:**

These results indicate that the antibacterial and antioxidant effects of EETC may contribute to oral hygiene products for dental caries management.

## 1 INTRODUCTION

Dental caries is an infectious bacterial disease (Caufield, 2005). Control of the number of bacteria in the oral cavity is very effective in preventing dental caries. Toothbrushing is a basic method to remove the dental plaque on the tooth surface, but it is difficult to effectively remove the plaque between the teeth with brushing only. Therefore, oral hygiene devices such as interdental toothbrush, dental floss, and gargling are used. In addition, antibacterial ingredients are used to effectively reduce the number of bacteria, but the problem of drug resistance is growing. In order to overcome these shortcomings, attempts to discover natural antibacterial ingredients for oral care are continuing.

The oral cavity is a suitable environment for bacterial growth and propagation. The presence of bacteria in the mouth readily stimulates the formation of dental plaque, which accumulates on both hard and soft tissues as dental calculus. The cause of tooth decay is the production of insoluble saccharide polymers and organic acids by oral bacteria. Glycosyltransferase (GTase) and fructosyltransferase (FTase) convert saccharide into insoluble polymers such as glucan and fructan (Wexler et al., 1993). These insoluble polymers adhere to the enamel surface and facilitates stable bacteria growth and incorporation of many types of bacteria. Taken together, locally attached bacteria produce persistent organic acids through glycolysis, which change the acidic environment around the tooth surface, thereby leading to demineralization. *Streptococcus* spp. and *Lactobacillus* spp. have been reported as being causative bacteria of tooth decay. *Streptococcus mutans* is one of the main causes of bacterial tooth decay. *S. mutans* is also an acid‐resistant bacteria and therefore contributes to sustained demineralization in acidic environments.

The antibacterial effect of *Terminalia chebula* fruit extracts have been reported on *Salmonella typhi*, *Staphylococcus epidermidis*, *Staphylococcus aureus*, *Bacillus subtilis*, and *Pseudomonas aeruginosa* in accordance with the extraction methods (Kannan et al., 2009). The antibacterial effect of *T. chebula* fruit extracts on *Streptococcus mutans* has been also reported (Nayak et al., 2014). However, our present understanding of the genetic mechanism in anticaries activity of the extract of *T. chebula* is insufficient. In addition to considering the antibacterial function of the extract of *T. chebula* on *S. mutant* in the oral cavity, we also need to investigate its safety on oral cells.

In the present study, we determined the antibacterial effects of an ethanol extract of *T. chebula* (EETC) on *S. mutans* and elucidated the biological mechanisms that support its anticaries effect. This result verifies that EETC is safe for gingival epithelial cells and effective in treating anticaries through antibacterial activity.

## 2 MATERIALS AND METHODS

### 2.1 Plant material

The ethanol extract of the *T. chebula* fruit was provided by COSMAX Inc. R&I Center (Seongnam, Korea). *T. chebula* fruit were collected from southwest China (Yunnan province). Taxonomic identification was done by a botanist and herbalist, Seok Kyun Yun, at COSMAX. The dried fruits were ground using an electronic miller. The powder was extracted using 70% ethanol for 72 h at room temperature, filtered through Whatman filter paper No. 1, and concentrated using a rotary evaporator under reduced pressure. The dried extracts were stored in a refrigerator until for further use. Stock solution was aliquoted and stored frozen at −70°C for up to 6 months. Freeze/thaw cycles were avoided.

### 2.2 Bacterial culture

*Streptococcus mutans* KCTC3065 was obtained from Korean Collection for Type Cultures (KCTC) in Korea Research Institute of Bioscience and Biotechnology (KRIBB) (Daejeon, Korea) and was cultured in brain‐heart infusion (BHI) broth (Becton, Dickinson and Company, Baltimore, MD, USA) or BHI agar at 37°C.

### 2.3 Drug susceptibility test

Drug susceptibility was assessed using the disc diffusion method. Briefly, a bacterial suspension in agarose solution was inoculated on BHI agar plates and the gel was allowed to solidify completely at room temperature. Whatman filter discs with infused drug were placed on the plates and cultured for 24 h. Drug susceptibility was assessed using a linear fitting of the squared radius (diameter in mm) of the inhibition zones.

### 2.4 MICs and MBCs

MICs for EETC was determined in triplicate via the broth dilution method after incubation at 37°C for 24 h. Briefly, EETC were added to 10 ml volumes of liquid medium resulting in 0, 0.1, 0.5, 1, 5, 10, 15, 20, 25, 30 μg/ml concentration and the cultured strain was inoculated such that the absorbance was 0.1 at 600 nm. After 24 h, the absorbance of the culture medium was measured at 600 nm. The concentration of EETC with an absorbance of 0.1 ± 001 or less was taken as the MIC of EETC. To determine the MBC for the EETC, bacteria were cultured for 24 h in a liquid medium containing a 10–10^−7^ serial dilution of EETC. 100 μl of the diluted bacterial solutions was smeared on agar plate and incubate for 3 days before counting the number of colonies. Then the number of bacteria per 100 μl was estimated by inverting the dilution factor and we defined the minimum concentration of the extract required to kill 99.99% of the bacterial cells compared to the control group to be the MBC.

### 2.5 Colony forming unit (CFUs)

The number of viable cells was measured using CFU. *S. mutans* was cultured until the absorbance of 600 nm was 0.4. 100 μl of the cultured medium was added to 900 μl liquid medium in which the EETC was added (0, 0.1, 0.5, 1, 5, 10, 15, 20 μg/ml) to make a 10‐fold diluted bacterial solution. Dilution was repeated to 10^−5^. 100 μl of the diluted bacterial solution was smeared on agar plates, incubated for 3 days, and colonies were counted.

### 2.6 Glucan formation

*S. mutans* was cultured in liquid media. The media was centrifuged at 10,000 rpm for 30 min and supernatant was filtered using 0.22 μm filter. After then, a filtrate was prepared using an Amicon Ultra Centrifugal Filter (MWCO 30 kDa, Cat. No. UFC903008, Millipore) and used as a bacterial enzyme solution containing GTase. 200 μl of enzyme solution and/or EETC was added to 800 μl of sucrose‐containing substrate solution [sucrose 12.5 mg, NaN_3_ 0.25 mg/ml of 50 mM potassium phosphate buffer (pH 6.5)] to make 1 ml reaction solution which were reacted at 37 °C for 36 h. Then reaction solution was centrifuged then and supernatant was discarded. 4 ml of 50 mM potassium phosphate buffer (pH 6.5) was added and sonicated for 5 min. The absorbance of this solution was measured at 550 nm.

### 2.7 RT‐PCR

Total RNA was isolated using the TRIzol reagent according to the manufacturer's instructions (Invitrogen, Carlsbad, CA, USA). First‐strand cDNA synthesis was performed using 2 μg of the total RNA and Promega's reverse transcription system (Madison, WI, USA). The primer sequence for RT‐PCR was shown in Table 1. PCR amplification was carried out in a reaction mixture containing 0.5 μg first‐strand cDNA and 10 pmol primers and consisted of 30 cycles. The amplified PCR product was electrophoresed on a 2% agarose gel in 1 × Tris‐Borate‐EDTA buffer containing ethidium bromide and visualized using the Gel Doc 2000 system (BioRad Laboratories, CA, USA). Images were analyzed using the Image J program in the same pixel area (National Institutes of Health, Bethesda, MD, USA).

**TABLE 1:** Primer sequence and annealing temperature for RT‐PCR

Target gene	Primer sequence
Glucosyltransferase B (*gtf B*)	Forward; 5'‐AGCAATGCAGCCAATCTACAAAT‐3' Reverse;5'‐ACGAACTTTGCCGTTATTGTCA‐3'
Glucosyltransferase C (*gtf C*)	Forward; 5'‐GGTTTAACGTCAAAATTAGCTGTATTAGC‐3' Reverse; 5'‐CTCAACCAACCGCCACTGTT‐3'
Glucosyltransferase D (*gtf D*)	Forward; 5'‐ACAGCAGACAGCAGCCAAGA‐3' Reverse; 5'‐ACTGGGTTTGCTGCGTTTG‐3'
Fructoxyltransferase (*ftf*)	Forward; 5'‐AAATATGAAGGCGGCTACAACG‐3' Reverse; 5'‐CTTCACCAGTCTTAGCATCCTGAA‐3'
GAPDH	Forward; 5'‐CCGCCTACTGCCCACTGCCACCAC‐3' Reverse; 5'‐TCCATCCACTATGTCAGCAGGTCC‐3'

### 2.8 DPPH radical‐scavenging activity

DPPH (2,2‐diphenyl‐1‐picrylhydrazyl hydrate) radical‐scavenging activity was determined by the method of Brand‐williams (Brand‐williams et al., 1995). 100 μl of 0.2 mM DPPH was mixed well with 20 μl of different dilution of EETC and reacted for 30 minutes in dark. Absorbance was measured at 517 nm with a microplate reader (Synergy™ HTX Multi‐Mode Microplate Reader, BioTek, Winooski, VT, USA). Ascorbic acid was used as positive control. The DPPH radical‐scavenging activity (%) was calculated by the following formular; (1 – [absorbance of the sample/absorbance of the control]) × 100.

### 2.9 ABTS‐radical‐scavenging activity

2,2‐Azino‐*bis*(3‐ethylbenzthiazoline6‐sulfonic acid) (ABTS) radical‐scavenging activity was determined by the ABTS‐radical cation decolorization assay (Re et al., 1999). 5 ml of 7 mM ABTS was mixed with 88 μl of 140 mM potassium persulfate and reacted for overnight in dark. Then reaction solution was diluted 5 mM phosphate buffer (pH 7.4) to 0.7 of 750 nm absorbance. 200 μl of diluted ABTS was mixed with 2 μl of different dilution of EETC and absorbance was measured at 750 nm after 6 minutes. The ABTS‐radical scavenging activity (%) was calculated by the following formular; (1 – [absorbance of the sample/absorbance of the control]) × 100.

### 2.10 MTT assay

Immortalized gingival fibroblasts were grown in DMEM/F12 (3:1) supplemented with 10% FBS, 1 × 10^−10^ M cholera toxin, 0.4 mg/ml hydrocortisone, 5 μg/ml insulin, 5 μg/ml transferrin and 2 × 10^−11^ M triiodothyronine at 37°C in a humidified atmosphere of 5% CO_2_. Cells (5 × 10^3^ cells/well) were seeded into 96‐well culture plates and left overnight to adhere. These cells were treated with various concentrations of EETC for 24 and 48 h, respectively. Viable cells were detected by incubating with 5 mg/ml MTT solution for an additional 4 h at 37°C, followed by the dissolution of the produced formazan product in the cells with 100 μl of DMSO. Absorbance was measured at 570 nm with a microplate reader (Synergy™ HTX Multi‐Mode Microplate Reader, BioTek).

### 2.11 Statistical analysis

Statistical analyzes were conducted using InStat GraphPad Prism ver. 5.01 statistical software (GraphPad Software, Inc., San Diego, CA, USA). Nonparametric Kruskal‐Wallis tests with Dunn's post hoc analysis was employed for multiple comparisons. The results are presented as the mean ± standard error of the mean (SEM). Asterisks were used to graphically indicate the statistical significance. Statistical significance was considered for *p* values of less than 0.05.

## 3 RESULTS

### 3.1 Antibacterial effect of EEDC on *S. mutans*

**FIGURE 1:**
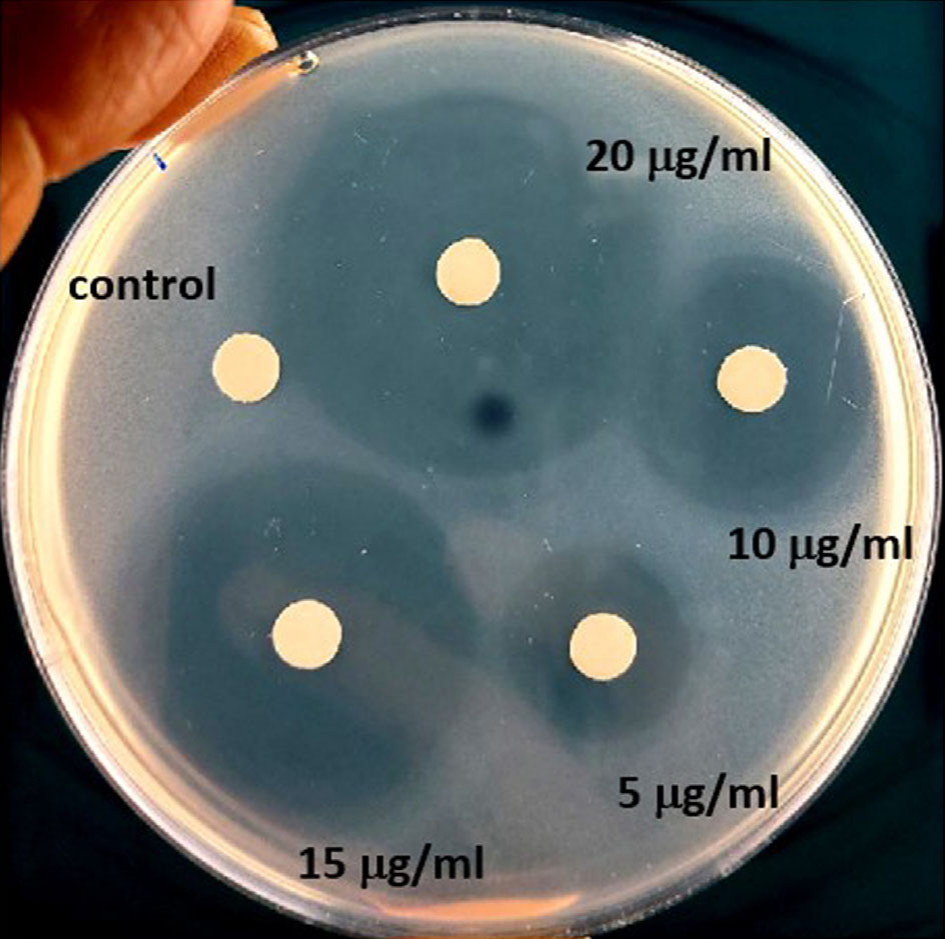
Antibacterial effect of EETC on *S. mutans* growth. *S. mutans* (1 × 10^4^ CFU) were cultured on an BHI agar plate with a paper disk containing the EETC. The diameter of the bacterial growth inhibition zone was calculated in millimeters (mm)

The disk diffusion method was used to verify the growth inhibition effect of the EETC on *S. mutans*. 1 × 10^4^ CFU of *S. mutans* were cultured on an agar plate with a paper disk containing different concentrations of EETC. As shown in Figure 1, 5 ~ 20 μg/ml EETC significantly inhibits *S. mutans* growth. The diameter of the clear zone where the *S. mutans* growth was suppressed is shown in Table 2, according to the concentration of EETC. We observed that a clear zone of 2.6 ± 0.23 mm at 5 μg/ml EETC, 6.03 ± 0.17 mm at 10 μg/ml, 10.21 ± 0.19 mm at 15 μg/ml, and 14.30 ± 0.11 mm at 20 μg/ml.

**TABLE 2:** Antibacterial activity of ethanol extract of *Terminalia chebula* (EETC) against *S. mutans*

Materials	Concentration (μg/ml)	Diameter of clear zone (mm)
Ethanol extract of *Terminalia chebula* (EETC)	0	0.00 ± 0.00
	5	2.6 ± 0.23*
	10	6.03 ± 0.17**
	15	10.21 ± 0.19**
	20	14.30 ± 0.11**

*Note*: *p < 0.05, **p < 0.001 versus without EETC.

### 3.2 Inhibitory effect of EETC on *S. mutans* growth

To observe the growth inhibition of EETC, *S. mutans* cultured in the 0 ~ 20 μg/ml EETC was diluted 10 times and then cultured on an agar plate to measure the number of colonies. As shown in Table 3, the number of colonies decreases depending upon the concentration of EETC. When the EETC concentration had been increased to 0, 1, 5, 10, 15, 20 μg/ml, the number of colonies decreased to 5.05, 4.98, 4.52, 1.79, 1.21, 0.24 log CFU/ml.

**TABLE 3:** Comparison of the CFU of the *S. mutans* at different concentrations of EETC

EETC Conc. (μg/ml)	0	1	5	10	15	20
Number of CFUs (log CFU/ml)	5.05	4.98	4.52*	1.79*	1.21*	0.24*

*p < 0.001 versus CFU from *S. mutant* culture without EETC.

### 3.3 MIC and MBC of *S. mutans* by EETC

In order to measure the MIC required for the EETC to inhibit the *S. mutans* growth, the absorbance was measured after culturing the *S. mutans* in a medium containing 0 ~ 30 μg/ml of EETC. In addition, *S. mutans* cultured in a medium containing EETC was diluted 10 times and cultured on an agar plate to count the number of colonies and measured MBC. As a result of this experiment, the MIC of *S. mutans* was 10 μg/ml and MBC was 20 μg/ml.

### 3.4 Inhibitory effect of EETC on glucan formation by *S. mutans*

**FIGURE 2:**
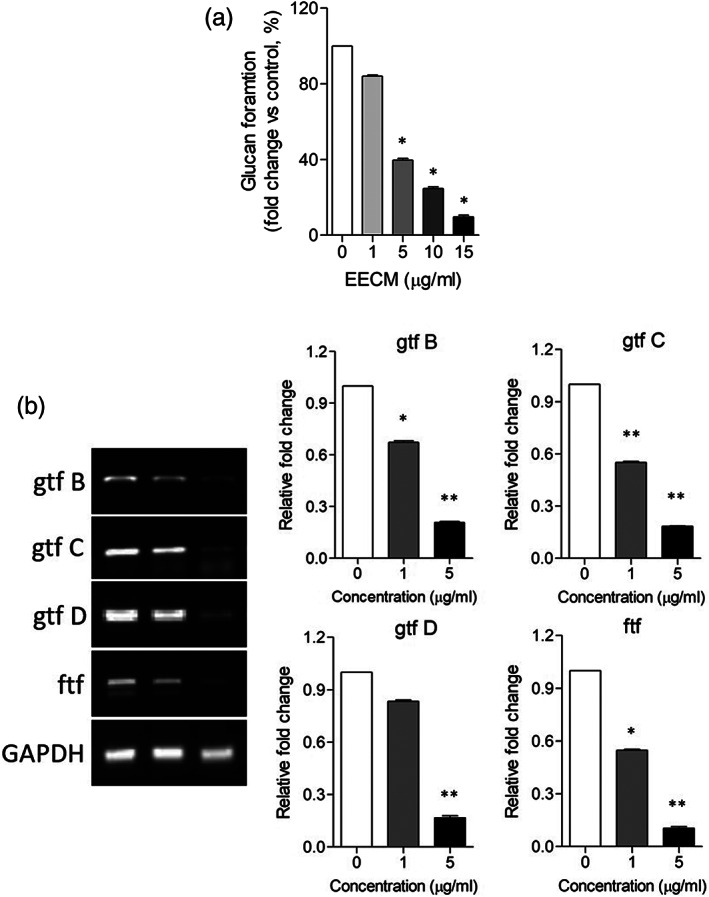
Inhibitory effect of EETC on glucan formation and gene expression related to insoluble saccharide polymer in *S. mutans*. (a) The filtrate of *S. mutans* culture medium and sucrose were mixed and kept for 36 h at 37°C. After centrifugation, the precipitate was sonicated and the absorbance was measured at 550 nm. *p < 0.001 versus reaction with the filtrate from *S. mutant* culture without EETC. (b) RT‐PCR was performed with primers for glycosyltransferase B (*gtf B*), glycosyltransferase C (*gtf C*), glycosyltransferase D (*gtf D*), and fructosyltransferase (*ftf*). Gene expression was observed in 2% agarose gel electrophoresis. The result shown is the representative images from several experiments. Images were captured and density was measured using ImageJ program. Relative density was plotted in fold changes as a graph. The values of the individual experiments are expressed as the mean ± standard error of three independent experiments. *p < 0.05, **p < 0.001 versus control

The glucan formation was analyzed in the reaction mixture with a filtrate of the *S. mutans* culture medium and sucrose as substrates. As shown in Figure 2a, significant glucan formation was observed in the reaction of sucrose and the *S. mutans* culture filtrate. However, EETC inhibited glucan formation in a dose‐dependent manner. When the EETC concentration was increased to 1, 5, 10, 15 μg/ml, the glucan formation decreased by 17, 60, 72, and 86%. Using RT‐PCR, we observed the expression of glycosyltransferase B (*gtf B*), glycosyltransferase C (*gtf C*), glycosyltransferase D (*gtf D*), and fructosyltransferase (*ftf*), which are involved in the formation of glucan and fructan. As shown in Figure 2b, 1 and 5 μg/ml of the EETC treatment significantly reduced the gene expression of the four enzymes. This result indicates that lower concentration than MIC of *S. mutans* by EETC inhibits glucan formation and related gene expression.

### 3.5 Effect of EETC on gingival fibroblasts

**FIGURE 3:**
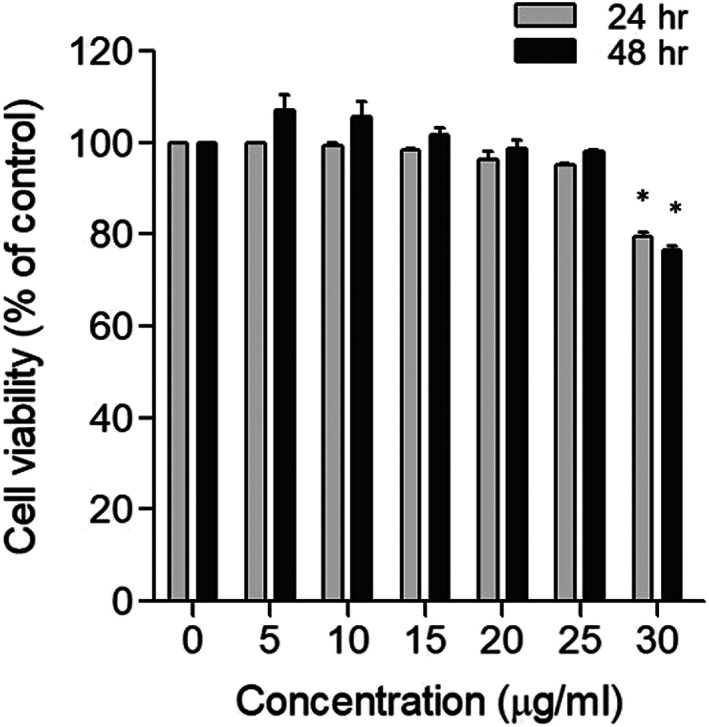
Effect of EETC on cytotoxicity. Gingival fibroblasts were cultured with or without EETC and live cells were analyzed using MTT assay. Cell viability is shown as % of control. The values of the individual experiments are expressed as the mean ± standard error of three independent experiments. *p < 0.05 versus control

The effect of EETC antibacterial concentration on gingival fibroblasts was analyzed by MTT assay. Cells were treated with 0 ~ 30 μg/ml of EETC for 24 and 48 h, and live cells were analyzed. As shown in Figure 3, we did not observe cytotoxicity below 25 μg/ml of EETC. However, a decrease of viable cells was observed at 30 μg/ml EETC. This result indicates that using MBC (20 μg/ml) of the EETC on *S. mutans* is safe for gingival fibroblast cells.

### 3.6 Antioxidant effect of EETC

**FIGURE 4:**
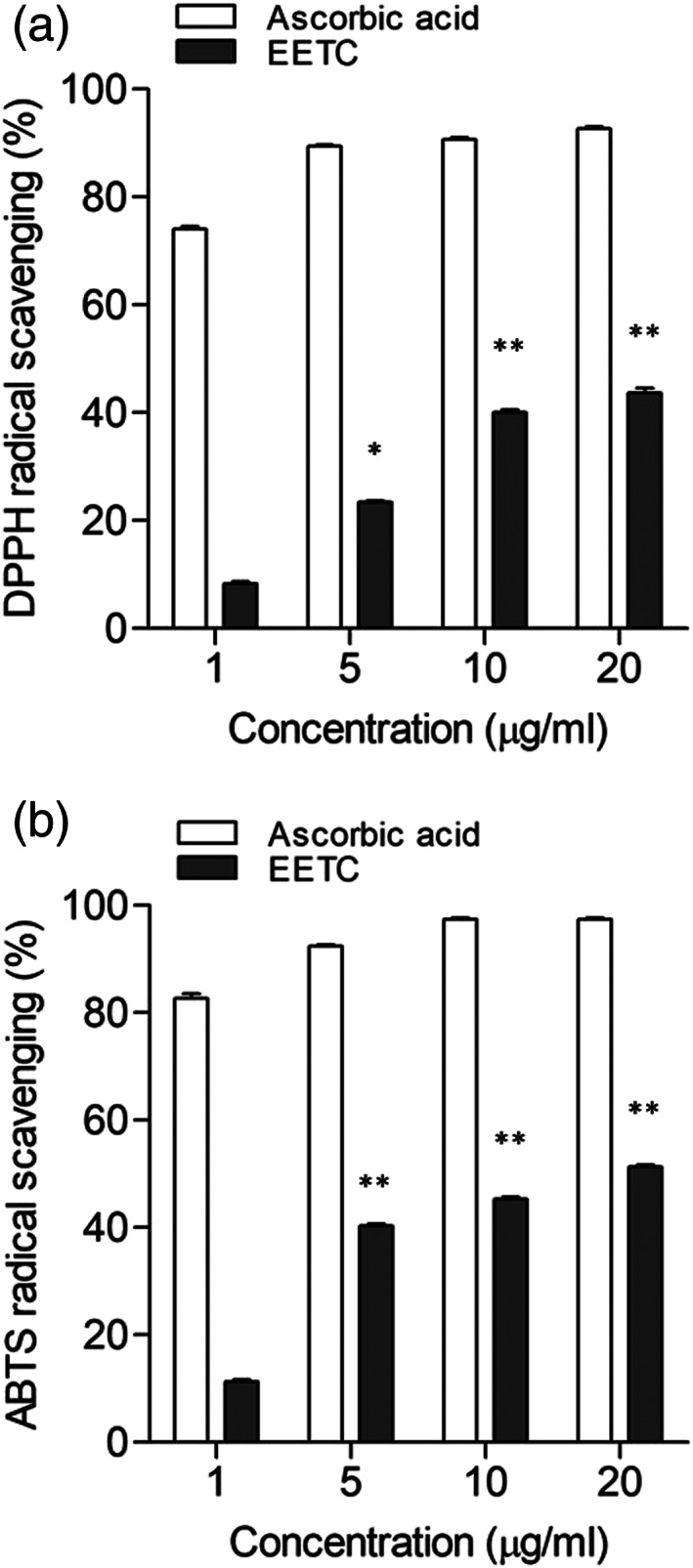
Antioxidant effect of EETC. (a) DPPH radical scavenging by EETC. (b) ABTS‐radical scavenging by EETC. Ascorbic acid was used as reductants control. The values of the individual experiments are expressed as the mean ± standard error of three independent experiments. *p < 0.01, *p < 0.001 versus 1 μg/ml EETC

To investigate the antioxidant effect of EETC, we tested its radical‐scavenging activity by using DPPH and ABTS free radicals. Ascorbic acid was used as a reductant for the radical‐scavenging molecule. As shown in Figure 4a,b, the radical‐scavenging activity of EETC was relatively lower than that of ascorbic acid, but it showed significant activity. The DPPH or ABTS‐radical‐scavenging activity of EETC was increased in a concentration‐dependent manner. The degree of DPPH radical scavenging of EETC was 23 ± 0.89% and 41 ± 1.23% at 5 and 10 μg/ml of EETC. The degree of ABTS‐radical scavenging by EETC was 39 ± 1.01% and 44 ± 1.36% at 5 and 10 μg/ml EETC.

## 4 DISCUSSION

According to the health care big data open system from the Health Insurance Review and Assessment Service in Korea, in 2010, the number of outpatients with dental caries (K02) was ranked seventh, with a total of 537 million, but in 2019, it ranked fourth with a total of 645 million. Along with the increase in the number of outpatients, medical care benefits for dental caries also increased from 2420 billion won in 2010 to 5397 billion won in 2019 (Statistics of Frequent Disease, 2019).

Dental plaque is a representative biofilm formed on the enamel surface. Oral bacteria attached to the pellicle of the tooth surface produce an extracellular substrate, such as glucan and fructan. They thereby form a structurally stable dental plaque by combining heterogeneous bacterial cells. Dental caries is caused by demineralization of the tooth surface by organic acids such as lactic acid, which is produced through sugar metabolism of bacteria. Enamel caries can progress to pulpitis through dentine caries, which can eventually lead to pulp infection and necrosis. *S. mutans*, *S. sobrinus*, *Lactobacillus casei*, *Lactobacillus viscosus*, *Lactobacillus acidophillus*, *Actinomyces viscosus*, and *Actinomyces odontolyticus* are mainly involved in dental caries (Struzycka, 2014). Particularly, markedly elevated levels of *S. mutans* is detected in the palque from the caries lesions and is considered to be the bacteria most closely contributes to the coronal decay (Duchin & van Houte, 1978.). *S. mutans* can produce organic acids through glycolysis, grows even in environments with a pH of 5.0 or less around the tooth surface biofilm, and maintains a significant glycolysis activity, thus exhibiting strong caries activity (Lemos & Burne, 2008). Facultative anaerobic bacteria *Lactobacillus casei*, *Lactobacillus viscosus*, *Lactobacillus acidophillus* are also acid‐resistant and produce lactic acid, and are particularly involved in caries progression such as dentine caries (Duchin & van Houte, 1978). *A. viscosus* and *A. odontolyticus* are implicated as the pathogen of root surface caries (Dame‐Teixeira et al., 2016).

Biofilm has a thick out layer formed by the insoluble polymer that outside molecules have difficulty penetrating. Bacterial interactions in biofilm easily induce genetic mutations, thereby increasing their resistance to antibiotics and antibacterial substances. Since the degree of deposition and maturity of dental plaques is related to the severity of oral diseases including dental caries, continuous management of dental plaques and maintenance of proper oral hygiene are important for the prevention of oral diseases. Long‐term use of synthetic drugs, such as antibiotics, for treatment of dental diseases causes antibiotic‐resistant bacteria. Therefore, for the purpose of preventing and treating oral diseases, there are active studies to observe the bacteriostatic or bactericidal effect of various plant extracts, including herbal medicines. In this study, we found that an ethanol extract of the *T. chebula* fruit (EETC) is effective in antibacterial use for dental caries that cause the bacteria *S. mutans*. The antibacterial activity of EETC was confirmed through disc diffusion analysis and CFU measurement. *Terminalia chebula* (myrobalan) is widely used in the traditional medicine of India and Iran to treat diseases that include dementia, constipation, and diabetes (Jokar et al., 2016). Many of these beneficial effects of *T. chebula* fruit are related to the presence of various phytochemicals, including steroids/sapogenins, saponins, anthraquinone derivatives, flavonoids, and tannins (Lee et al., 2007; Rathinamoorthy & Thilagavathi, 2014). The most important component in the fruit is tannin. *T. chebula* has a tannin content of 32%–45% that includes gallic acid, ellagic acid, chebulic acid, chebulinic acid, punicalagin, and tannic acid. The flavonoids quercetin, catechin, and kaempferol have been detected (Jokar et al., 2016). *T. chebula* fruit is effective in the treatment of bacterial infections (Kim et al., 2006; Rai & Radhika, 2009). Clinical trials of *T. chebula* fruit extract as a mouthwash preparation have been reported to reduce plaque accumulation and gingival inflammation (Gupta et al., 2015; Naiktari et al., 2014). It will be necessary to isolation the pure compound from the extract to identify the components, according to their medical value.

We also provide the mechanistic details for anticaries effect of EEDC in this study. Since many studies on the development of natural antibacterial agents have focused only on bacteriostatic and bactericidal analysis, biochemical and genetic analysis are very poor. This limits the data for understanding the antibacterial mechanism. The three characteristics that closely relate *S. mutans* to dental caries are (1) they form extracellular polymers such as glucan using sugars, (2) they contribute to the permanent colonization of hard surfaces containing various bacteria, and (3) they produce organic acids through glycolysis and even survive at low pH (aciduricity) (Lemos & Burne, 2008). *S. mutans* produces three glucosyltransferases (GTFs), mainly, *gtf‐B*, *gtf‐C* and *gtf‐D*. These utilize the glucose moiety of sucrose as a substrate to synthesize glucose polymers of glucans through *α*(1–3)‐ and *α*(1–6)‐linkages, and the fructose moiety of sucrose produces lactic acid through glycolysis (Lemos & Burne, 2008). Fructosyltransferases (FTFs) of *S. mutans* also catalyze the production of fructose polymers of fructans. Fructans serve as short‐term extracellular carbohydrate sources and are degraded by the fructanase enzyme FruA, yielding fructose, which can be internalized for energy production. They also produce organic acid. Therefore, GTFs have a pivotal virulence in dental caries through their contribution to biofilm buildup by acid production and forming a glue‐like polysaccharide matrix. In this study, glucan formation was observed in the reaction of sucrose and *S. mutans* culture filtrate, and EETC treatment significantly reduced glucan production. To investigate mechanistic details for extracellular insoluble polymers, we performed RT‐PCR. Increasing the EETC concentration significantly reduced the gene expression of glycosyltransferase B (*gtf B*), glycosyltransferase C (*gtf C*), glycosyltransferase D (*gtf D*), and fructosyltransferase (*ftf*). Even at the 10 μg/ml EETC for MIC of *S. mutans*, we observed the remarkable inhibition of the enzymes producing insoluble polymers. In addition, ≤25 μg/ml of EETC was verified to be noncytotoxic in human gingival epithelial cells.

In the present study, we elucidated the antioxidant effect of EETC with DPPH and ABTS‐radical scavenging assays. Free radicals are highly reactive with other cellular structures because they contain unpaired electrons. Therefore, free radicals can damage the tissues and cells by stealing their electrons through oxidative reaction. Various external stimuli and inflammation conditions, such as gum disease, increase oxidative stress in the oral mucosa. High concentrations of reactive oxygen species (ROS) are produced at the plasma membrane in the vicinity of such pathogen as *Streptococcus sanguinis* and *Streptococcus gordonii* (Touati, 2000). *S. mutans* can produce ROS in vitro (Fujishima et al., 2013; Wang et al., 2001). Therefore, it is very important to find the antioxidants for scavenging these free radicals. From the standpoint of in vitro methods for assessing antioxidative activities, several have been proposed for evaluating them by using 1,1‐diphenyl‐2‐pierylhydrazyl (DPPH) and 2,2′‐azino‐*bis*‐(3‐ethylbenzo thiazolin‐6‐sulfonic acid) diammonium salt (ABTS). DPPH or ABTS‐radical‐scavenging activity of EETC was increased in a concentration‐dependent manner. Particularly, antioxidant activity was observed at lower concentration (≥10 μg/ml) than MIC of the EETC on *S. mutans*. This result indicates that the EETC can be applied as an antibacterial and antioxidant regardless of cytotoxicity.

In conclusion, this study elucidated that the EETC has effective anticaries activity through the inhibition of glucan formation and related gene expression, as well as antibacterial effects under low cytotoxic concentrations of EETC. Therefore, EETC could be applied to oral hygiene products, with its antibacterial properties, for dental caries management. Further studies are required to isolate which pure compounds are related to antibacterial activity. Clinical or experimental animals studies will be needed to verify the anticariogenic effect of EETC.

## CONFLICT OF INTEREST

The authors have no conflict of interest to disclose.

## Data Availability

The data support the findings of this study are available from the corresponding author upon reasonable request.
